# Long term follow up after surgery for benign intracranial cysts in children

**DOI:** 10.1186/2045-8118-12-S1-P41

**Published:** 2015-09-18

**Authors:** Katrin KM Rabiei, Roberto Doria Medina, Mats Högfeldt, Carsten Wikkelsö, Magnus Tisell

**Affiliations:** 1 Hydrocephalus research unit, Institution of Neuroscience, Sahlgrenska Academy, Sweden

## 

Arachnoid cysts are cystic malformations in cerebrospinal axis found in both adults and children. While most arachnoid cysts are asymptomatic and usually go undetected, some cause symptoms and warrant surgical treatment. In this prospective study we aimed to describe the result of short- and long term follow up in children referred to our center with a cystic malformation.

## Methods

27 pediatric patients (13 f, 14m, mean age 9, 4 y) with de-novo cysts were consecutively included during a 5 year period. Reason for initial investigation was headache, seizures, endocrine dysfunction, macrocephaly, balance disturbance and/or dizziness, trauma, cognitive disturbance and syncope. 22 patients underwent surgical treatment after initial evaluation with either open- or endoscopic fenestration of the cyst wall. Cyst volume was measured pre- and postoperatively with OsiriX software. Short term follow up was conducted 3 months and long term follow up 8, 6 years (7-10, 5 y) postoperatively.

## Results

60% (13/22) of the patients were improved after the short term - and 82% (18/22) after the long term follow up considering at least one major complaint. Operated cysts had a mean preoperative volume of 60 ml (5-225 ml) which postoperatively reduced with average of 56% at 3 month follow up. There was no significant difference in postoperative cyst volume between patients who improved and those who did not. Headache and imbalance improved significantly in the long-term follow up but not in the short term. Some individuals improved in Cognitive function, seizures and endocrine dysfunction. There was no permanent postoperative morbidity.

## Conclusion

82% of the children operated improved in the long-term follow up. Clinical improvement did not correlate with radiological improvement.
